# Surgical management of biliary fistula following associating liver partition and portal vein ligation for staged hepatectomy in pediatric hepatoblastoma: a case report and literature review

**DOI:** 10.3389/fonc.2025.1700516

**Published:** 2025-12-15

**Authors:** Gang Shen, Yunpeng Zhai, Huashan Zhao, Rui Guo, Hongxiu Xu, Sai Huang, Shisong Zhang

**Affiliations:** Department of Thoracic and Oncology Surgery, Children’s Hospital Affiliated to Shandong University (Jinan Children’s Hospital), Jinan, China

**Keywords:** hepatoblastoma, ALPPS, biliary fistula, case report, surgery

## Abstract

**Objective:**

To report the clinical data of a pediatric patient with massive hepatoblastoma who underwent associating liver partition and portal vein ligation for staged hepatectomy (ALPPS) following chemotherapy.

**Case presentation:**

A 1-year-and-8-month-old male infant was diagnosed prenatally with an intrahepatic mass, which was later confirmed as hepatoblastoma via ultrasound-guided biopsy postnatally. The parents declined surgical intervention, leading to voluntary discharge. The child received oral lenvatinib therapy externally, but the tumor continued to enlarge (13.1cm × 7.9cm × 9.9cm), prompting hospital admission. Four cycles of C5VD chemotherapy (cisplatin + vincristine + 5-fluorouracil) were administered, resulting in limited tumor reduction (11.3cm × 5.6cm × 10.8cm). Minimal shrinkage was observed after the fourth cycle compared to the second, with significant intratumoral calcification, suggesting suboptimal further chemotherapeutic efficacy. Surgical intervention was thus decided. Preoperative assessment indicated that direct tumor resection would leave only 24% of the future liver remnant, posing a high risk of liver failure. ALPPS was consequently selected. During the first stage (February 25, 2025), liver partition and right portal vein ligation were performed. Ten days postoperatively, FLR increased to 35%, enabling the second-stage tumor resection (March 9, 2025). Postoperative biliary fistula developed at the liver transection plane and failed conservative management, necessitating a third procedure (hepaticojejunostomy via Roux-en-Y anastomosis) for successful biliary-enteric drainage reconstruction. Adjuvant chemotherapy was continued postoperatively, with no recurrence observed during follow-up.

**Conclusion:**

ALPPS is a safe and effective surgical approach for pediatric patients with massive hepatoblastoma requiring extensive resection and presenting with insufficient FLR.

## 1 Introduction

Hepatoblastoma (HB) is the most common primary malignant liver tumor in children, accounting for approximately 80% of pediatric liver malignancies, the incidence rate among children is approximately 1.5 cases per million children per year. Surgical resection remains the cornerstone treatment for hepatoblastoma; however, complete resection is unfeasible in 50%-60% of initial presentations due to large tumor size or invasion of major vascular structures (1). For such complex cases, the associating liver partition and portal vein ligation for staged hepatectomy (ALPPS) technique—a method promoting rapid regrowth of the residual liver volume—offers the possibility of achieving curative tumor resection (2). Yet, postoperative biliary fistula remains a common and severe complication of ALPPS, occurring in 14%-20.63% of cases. The risk significantly increases in procedures involving biliary reconstruction or extensive hepatectomy (3).

The etiology of biliary fistula is complex, potentially related to intraoperative biliary tract injury, improper management of the hepatic margin bile duct, or biliary ischemia following portal vein ligation (4). In pediatric hepatoblastoma, the intricate hepatic anatomy and frequent tumor invasion of the hepatic hilum further complicate surgery. While existing literature extensively discusses management of biliary fistula after adult hepatobiliary surgery, reports on ALPPS-associated biliary fistula in pediatric hepatoblastoma are scarce, with no consensus on risk factors, prevention strategies, or management protocols. Furthermore, cytokines released during liver regeneration after resection may promote residual tumor growth (5), making timely control of biliary fistula particularly critical for prognosis. This report details a case of biliary fistula following ALPPS for pediatric hepatoblastoma. By analyzing its pathogenesis, clinical management, and prognostic implications in conjunction with the literature, we aim to provide guidance for preventing and managing this complex complication.

## 2 Case report

### 2.1 Preoperative status

**Figure 1:**
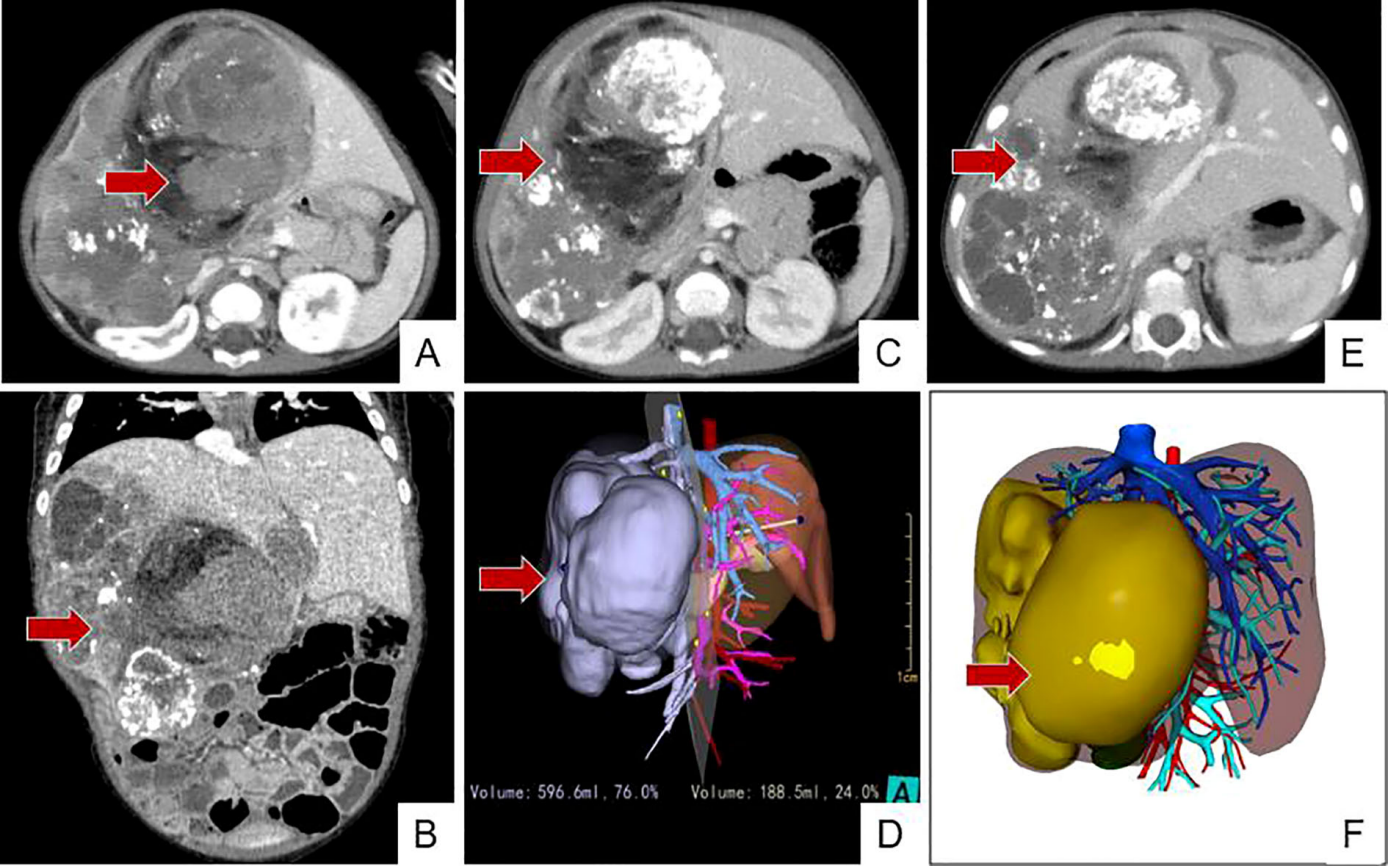
**(A, B)** Pre-chemotherapy CT. A massive tumor is visible within the liver parenchyma, measuring approximately 13cm × 10cm × 8cm overall and invading the entire right triad, three-dimensional reconstruction simulation surgery indicates that the residual liver volume after tumor resection is 24%. **(C, D)** After four cycles of chemotherapy, the tumor has slightly reduced in size but still occupies the right triad. **(E, F)** Preoperative images for the second surgery. The tumor remains large, with slight enlargement of the left lateral lobe. (Arrows indicate tumor).

A male patient, 1 year and 8 months old at admission. A hepatic tumor was detected during the mother’s prenatal examinations. At 3 months postnatal, a liver biopsy revealed hepatoblastoma with extramedullary hematopoiesis (embryonal type). The parents declined surgery and were discharged, subsequently administering lenvatinib orally at an external facility. Due to marked abdominal enlargement compared to previous measurements, the patient was admitted to our hospital on November 10, 2024. Enhanced abdominal CT revealed multiple large, heterogeneous-density masses of varying sizes within the hepatic parenchyma. The overall size of the lesions approximately 13.1 cm × 7.9 cm × 9.9 cm, with a larger mass on the left side. Multiple nodular and patchy calcifications were noted at the margins and within the lesions. The lesions exhibited heterogeneous density with multiple cystic-like low-density areas. Contrast-enhanced imaging demonstrated marked heterogeneous enhancement (**Figures 1A, B**). Due to the large tumor size, surgery was not feasible. After ruling out contraindications, the patient received the first course of C5VD chemotherapy (cisplatin + 5-fluorouracil + vincristine + doxorubicin). The chemotherapy process proceeded smoothly. Post-chemotherapy complications included bone marrow suppression and febrile infection, treated with human granulocyte colony-stimulating factor (G-CSF), meropenem, and fluconazole. Following two cycles of C5VD chemotherapy, an abdominal contrast-enhanced CT revealed the tumor lesion measuring approximately 12.1 cm × 6.6 cm × 10.9 cm. The tumor remains large, with a small residual liver volume post-surgery. Additionally, the tumor is closely adherent to the inferior vena cava, making it impossible to achieve R0 resection during surgery. As a result, surgery remains unfeasible, and it was decided to continue chemotherapy (6). After two additional cycles of C5VD chemotherapy, another abdominal contrast-enhanced CT showed the lesion measuring approximately 11.3 cm × 5.6 cm × 10.8 cm, with increased intratumoral calcifications and a slight reduction in lesion volume (**Figures 1C, D**). Considering the slow tumor shrinkage after four cycles of chemotherapy, surgical intervention was decided.

### 2.2 ALPPS surgical approach

Preoperative 3D CT reconstruction indicated that complete tumor resection would leave only 24% of the liver volume intact, posing a high risk of hepatic failure. Because during the operation, a small portion of liver tissue adjacent to the tumor may be resected as well, if a right trisegmentectomy is performed through a conventional one-stage hepatectomy, the volume of the residual liver will be too small after the operation. Therefore, a staged approach was chosen: a primary procedure involving hepatic division and ligation of the right portal vein branch, followed by a secondary hepatoblastoma resection once the remaining liver volume had increased.

**Figure 2:**
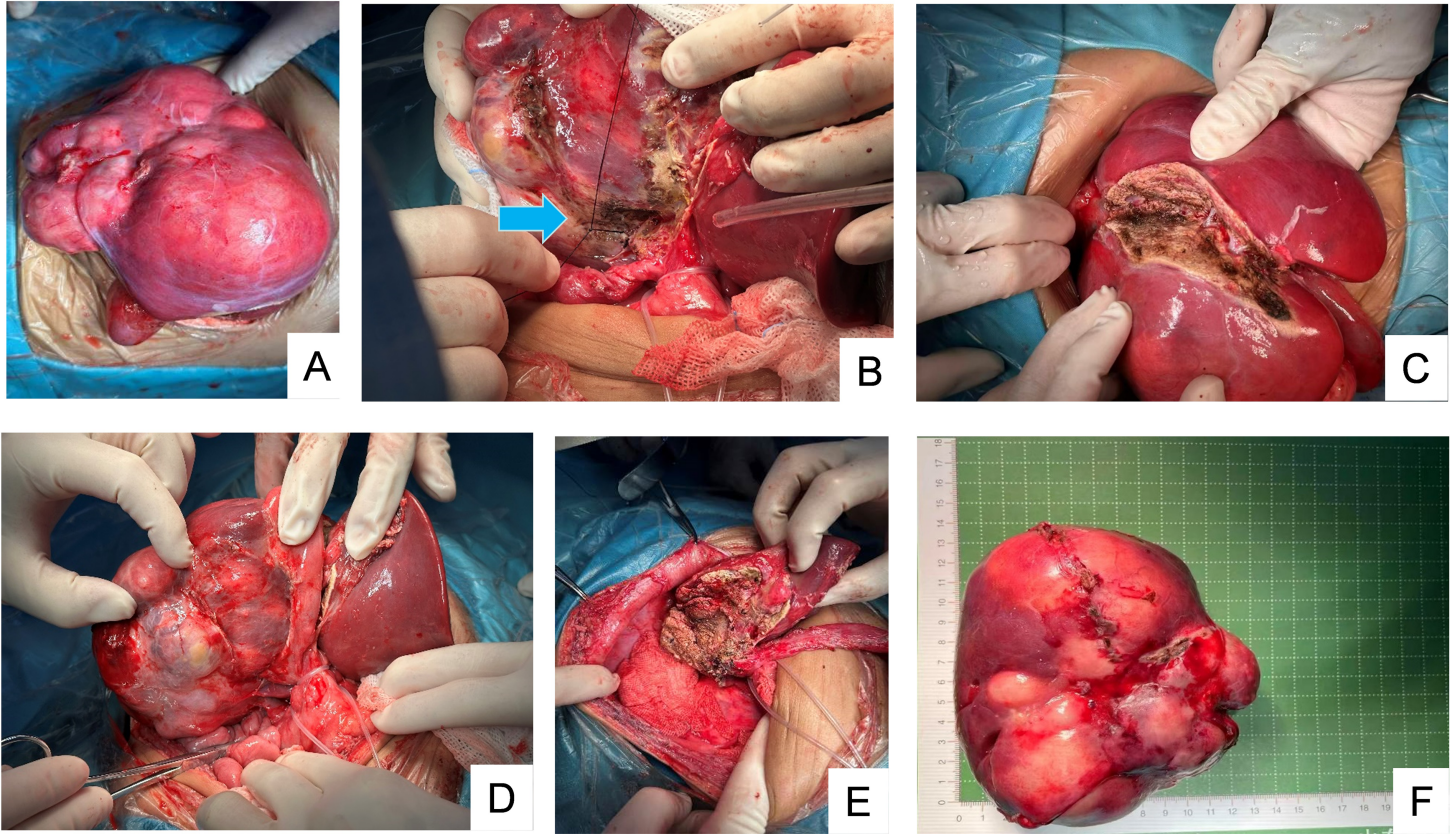
**(A–C)** Findings from the first surgery. **(A)** Hepatic tumor occupying the right hepatic triad; **(B)** Ligation of the right portal vein branch; **(C)** Dissection of the liver along the falciform ligament. **(D–F)** Findings from the second surgery. **(D)** Tumor dissection; **(E)** After complete tumor resection; **(F)** Overall appearance after tumor removal.

The first-stage surgery was performed on February 25, 2025 (**Figures 2A–C**). A 30-cm midline incision was made 1 cm below the right costal margin in the right upper abdomen. The primary tumor involved three lobes of the right liver, measuring approximately 12 cm × 11 cm × 6 cm. It was solid in consistency with rich vascular supply. The peritoneum anterior to the common bile duct was opened to expose the proximal common bile duct. The cystic duct was dissected, and liver tissue was separated superior to both the cystic duct and common bile duct to expose the right branch of the portal vein, which was ligated with two 2–0 silk sutures. Intraoperative color Doppler ultrasound confirmed uncompromised blood supply to the left portal vein branch. The left lateral lobe showed no tumor involvement. Along the tumor margin, dissection was performed anteriorly from the anterior margin of the Rex fossa and posteriorly to the inferior vena cava, excising the hepatic tissue between the three right hepatic lobes and the left lateral lobe. The procedure was completed.

A follow-up CT scan with 3D reconstruction performed 10 days postoperatively showed the residual liver volume increased to 35% after tumor resection (**Figures 1E, F**). Consequently, a second-stage hepatoblastoma resection was performed on March 9, 2025. Access was gained through the original incision. The hepatic hilum was clamped with a tourniquet, and dissection was performed at the hilum. The right branch of the portal vein, the right branch of the hepatic artery, and the common hepatic duct were ligated. Dissection continued along the tumor margin, following the previous resection plane, and the right branch of the middle hepatic vein and the right hepatic vein were transected. While ensuring negative resection margins, dissection along the tumor margin was performed using a microwave knife. Hepatic ducts, arteries, and veins entering the tumor were ligated and sutured during this process to achieve complete hemostasis. The hepatic tumor tissue was completely excised, and the gallbladder was preserved and sutured to the left hepatic lobe. Hepatic portal blood flow was occluded, and Methylene Blue Injection was injected through the gallbladder. Good blue staining of the hepatic tissue surface was observed, with punctate methylene blue leakage visible on the hepatic resection margin. Leakage points were meticulously identified and ligated; no further leakage was detected upon re-examination. Intraoperative color Doppler ultrasound confirmed no residual tumor, and no abnormalities of the bile duct were found either. The procedure concluded with placement of a peritoneal drainage tube. (**Figures 2D–F**).

### 2.3 Postoperative course

On postoperative day 2, the patient developed icterus of the sclera and skin. Total bilirubin increased to 174.8 μmol/L, direct bilirubin rose to 139.7 μmol/L, and abdominal drainage yielded 240 mL of pale red fluid. On postoperative day 3, scleral and cutaneous jaundice slightly improved. Total bilirubin was 76.8 μmol/L, direct bilirubin was 61.8 μmol/L, and abdominal drainage yielded 230 mL of pale yellow bile-stained fluid, indicating biliary fistula formation. Subsequently, the patient’s total and direct bilirubin gradually returned to normal levels, with abdominal drainage ranging from 100–200 ml. Despite treatment including anti-inflammatory agents, fluid replacement, nutritional support, and albumin administration, the biliary fistula persisted without improvement. Consequently, a third surgical intervention was planned.

**Figure 3:**
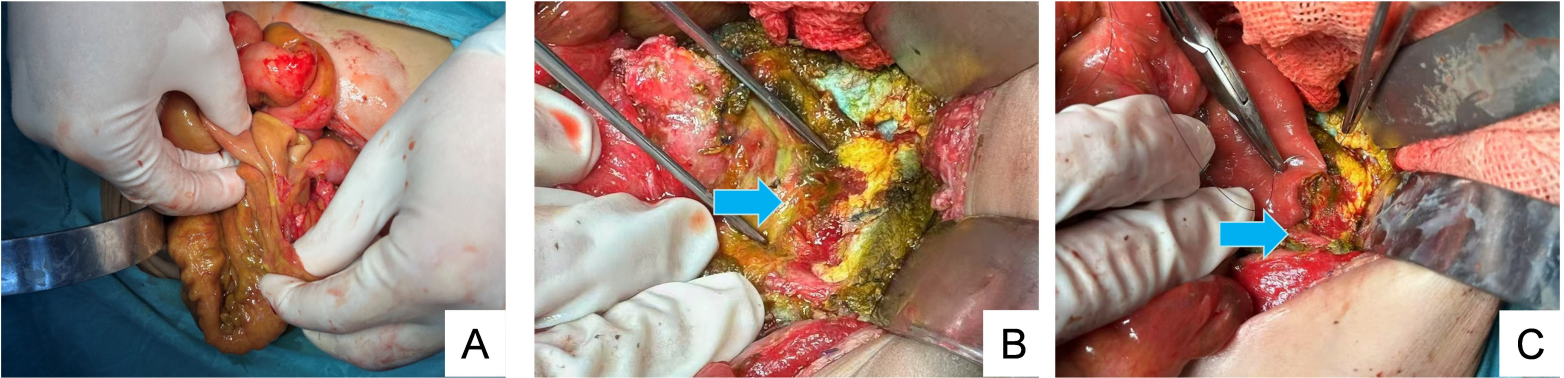
**(A–C)** Third surgical procedure. **(A)** Intestinal surfaces covered with extensive yellow bile-like pus; **(B)** Three bile duct fistulas visible on the liver cut surface (arrows); **(C)** Hepatojejunal anastomosis (indicated by arrow).

The procedure was performed on March 25, 2025. Access was gained through the left half of the original incision. The abdominal cavity contained a significant amount of yellow-green bile, and the intestinal surfaces were covered with multiple yellow bile-like purulent crusts. Adhesions were gently separated. The gallbladder was markedly edematous and contained white bile. The gallbladder and cystic duct were dissected, and the gallbladder was resected with the cystic duct sutured. Examination of the hepatic stump revealed three bile duct openings leaking bile, spaced approximately 1 cm apart, with a duct diameter of about 0.2 cm. A definitive diagnosis of biliary fistula was made, leading to the decision for a hepaticojejunal Roux-en-Y anastomosis. The jejunum was transected 15 cm distal to the Treitz ligament, and the distal intestinal end was closed. A 30 cm lateral incision was made on the distal jejunal segment, which was end-to-side anastomosed to the proximal jejunal stump. The mesenteric orifice was sutured closed. The jejunal segment serving as the biliary bypass was retracted from behind the transverse colon to the choledochal fistula site, and the mesenteric orifice was sutured closed. Approximately 2 cm distally from the anastomotic end, a 2 cm longitudinal incision was made in the jejunal segment. This segment was sutured to the hepatic tissue surrounding the choledochal fistula site (end-to-end mucosal-to-mucosal anastomosis). No additional bile fistula sites were identified. A single abdominal drainage tube was placed. The procedure was completed. (**Figure 3**).

**Figure 4:**
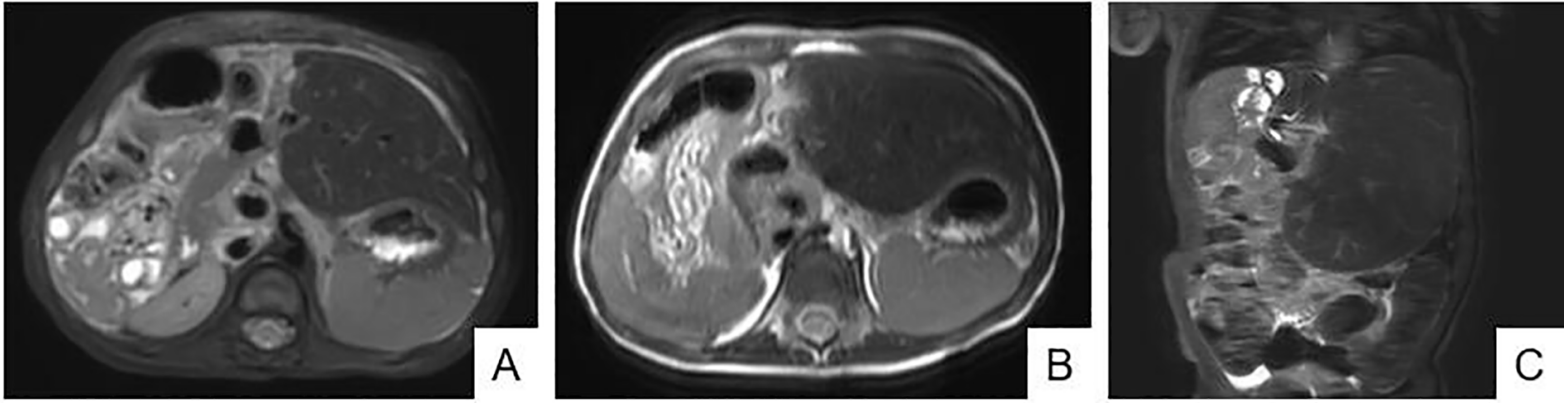
Postoperative Follow-up MRI. Follow-up results at 1 month **(A)** and 6 months **(B, C)** postoperatively show the remaining liver is well-preserved, with no tumor recurrence or metastasis to other sites.

Postoperatively, the patient continued anti-inflammatory therapy, fluid resuscitation, nutritional support, albumin administration, and other treatments. The patient recovered well from surgery and subsequently underwent four cycles of C5VD chemotherapy. After six months of postoperative follow-up and re-examination, no recurrence or metastasis was observed (**Figure 4**).

## 3 Discussion

Hepatoblastoma (HB), as the most common primary malignant liver tumor in children, remains primarily treated by surgical resection. However, for PRETEXT III or IV stage tumors invading the hepatic hilum or cases with insufficient residual liver volume, an alternative approach is the ALPPS. The case reported herein successfully achieved resection of a POST-TEXT stage III tumor via ALPPS technique, though postoperative biliary fistula complications warrant further discussion.

Advances in surgical management of POST-TEXT III and IV hepatoblastomas primarily involve innovative surgical approaches and optimized multidisciplinary strategies. For locally advanced tumors, traditional hepatectomy faces technical limitations, prompting recent developments such as the two-stage ALPPS procedure. Literature indicates that for PRETEXT III cases involving hilar invasion, tricuspid resection following portal vein embolization (PVE) achieves R0 resection with manageable complications (7). Furthermore, the introduction of precision resection concepts enables preservation of greater functional liver parenchyma, particularly beneficial for pediatric patients. For POST-TEXT IV or vascular invasion (POST-TEXT V+) cases, comparative studies between liver transplantation (LT) and extended resection indicate both approaches can achieve 5-year survival rates of 80-90% under specific conditions, though selection must be individualized based on tumor biology (8). Notably, preoperative chemotherapy significantly improves the convertibility rate of unresectable tumors, but its toxic side effects must be balanced against surgical timing. Regarding postoperative complication management, studies emphasize the need for particular attention to risk factors such as preoperative tumor rupture and distant metastasis, which are significantly associated with poor prognosis. For tumor thrombi involving major vascular systems (e.g., inferior vena cava or portal vein trunk), multicenter data confirm the safety and feasibility of surgical resection combined with vascular reconstruction, with a postoperative recurrence rate of approximately 33.3% but no perioperative mortality (9). Although large-scale reports on repeated hepatectomy in recurrent cases are scarce, preliminary data suggest it may prolong survival. International collaborative groups (e.g., SIOPEL and IPSO) are advancing technical consensus through standardized surgical surveys, while the POST-TEXT staging system combined with CHIC-HS risk stratification further optimizes surgical decision-making (10). Overall, technical advances have reduced the proportion of extreme hepatectomies, but vigilance is warranted regarding the positive correlation between operative time and complications.

The ALPPS procedure (two-stage hepatectomy combining liver dissection and portal vein ligation) is an innovative surgical technique for treating unresectable liver tumors, increasingly applied in pediatric hepatoblastoma management in recent years. Its core principle involves achieving rapid and significant functional liver reserve (FLR) regeneration through a two-stage procedure, enabling resection of tumors previously unresectable due to insufficient residual liver volume (11) and thereby expanding the indications for tumor resection. The first stage involves ligating the affected portal vein branch to redirect blood flow to the healthy hepatic lobe. This is combined with liver parenchymal transection (typically along the ischemia line) to block the formation of collateral circulation within the liver, thereby accelerating compensatory hyperplasia in the healthy lobe. Studies indicate ALPPS achieves 40-80% FLR growth within 7–10 days, with a proliferation rate 2–3 times faster than conventional portal vein embolization (PVE) (12). Following Phase I, tumor resection proceeds in Phase II only after imaging confirms FLR volume meets the target (typically 25-30% of standard liver volume in children).

Key indications for ALPPS in pediatric hepatocellular tumors include: (1) Initially unresectable central hepatoblastoma: Particularly PRETEXT II-III stage tumors where conventional surgery cannot guarantee R0 resection and residual liver volume is insufficient. Literature reports ALPPS as an alternative to liver transplantation, enabling successful tumor removal while avoiding transplant-related complications and donor shortages (11). (2) Large-volume or vascular-invasive tumors: ALPPS enables radical resection of rare tumors such as rhabdomyosarcomas invading all three hepatic veins (13). (3) Cases with limited resection due to chemotherapy-induced liver injury: ALPPS improves surgical safety by promoting rapid regeneration in children with diminished hepatic reserve following chemotherapy (14).

In pediatric hepatoblastoma, ALPPS is particularly suitable for centrally located PRETEXT II or III tumors, which traditionally may require liver transplantation. Studies indicate that ALPPS enables extensive hepatectomy by inducing rapid regeneration of healthy liver tissue, thereby avoiding the high costs, donor shortages, and postoperative complications associated with transplantation (10). Furthermore, ALPPS has demonstrated feasibility in tumor centers within low- and middle-income countries. Despite its high technical demands, strict case selection enables favorable treatment outcomes (15).

Biliary fistula is a common postoperative complication of hepatectomy, potentially leading to postoperative infection, liver failure, or even death. Its occurrence is associated with multiple factors, including surgical technique, bile duct injury, extent of liver resection, and postoperative management strategies (16).

In pediatric hepatoblastoma surgery, key risk factors for biliary fistula include extensive hepatectomy (e.g., hemihepatectomy or extended hemihepatectomy) and intraoperative bile duct injury. For instance, one study reported that biliary fistula in 6 pediatric patients undergoing liver tumor surgery was closely associated with intraoperative bile duct injury, with some cases requiring reoperation for bile duct repair (17). Additionally, bile fistula development is associated with factors such as inadequate postoperative bile drainage and biliary anastomosis techniques (18).

Treatment approaches for bile fistulas encompass conservative management (e.g., drainage, antibiotic use) and invasive interventions (e.g., surgical repair or endoscopic procedures). For isolated bile fistulas, most cases require surgical repair, particularly when the fistula persists or is complicated by bile duct stricture (16). In recent years, minimally invasive techniques such as percutaneous transhepatic bile duct drainage (PTBD) have demonstrated favorable outcomes in managing postoperative bile fistulas following pediatric liver transplantation, effectively reducing bile leakage and promoting fistula closure (19). Additionally, indocyanine green (ICG) fluorescence imaging has been employed for intraoperative bile duct localization and early postoperative fistula diagnosis, contributing to reduced fistula incidence (20).

During the initial surgery, we injected Methylene Blue Injection into the gallbladder and observed leakage at the liver incision site. Subsequently, we addressed the leakage by ligation, but did not re-examine the site for leakage. Combined with the intraoperative finding of white bile in the gallbladder, it is speculated that the postoperative bile leak in this case resulted from intraoperative injury to the bile duct and erroneous ligation of the common bile duct. This directly obstructed bile drainage, subsequently leading to the bile leak. Literature indicates that erroneous ligation of the common bile duct represents a rare yet severe cause of postoperative cholangitis following hepatobiliary surgery, with heightened risk in cases of anatomical variation or inadequate surgical exposure (21). Related studies suggest that elevated bile duct pressure following ligation may cause microbile duct rupture, potentially leading to secondary biliary peritonitis (22). The intraoperative findings in this case align with the mechanism of iatrogenic bile duct injury described in the literature, namely failure to clearly identify the anatomical structure of the common bile duct during surgery, leading to misjudgment of the ligation site. This lesson underscores that in complex procedures involving combined hepatic resection and portal vein ligation, it is advisable to confirm bile duct course using intraoperative cholangiography or fluorescence imaging techniques, supplemented by intraoperative ultrasound when necessary, to prevent accidental injury.

During the hepatectomy, we preserved the gallbladder primarily because the tumor had not invaded it, the gallbladder retained its bile storage function, and gallbladder preservation may reduce certain postoperative biliary complications (23). However, during the third surgery, the gallbladder was found to be markedly edematous and contained white bile. Given the diminished gallbladder function, it was subsequently resected.

Preventing such complications hinges on optimizing surgical protocols: First, for procedures requiring extensive hepatoduodenal dissection (e.g., pediatric hepatoblastoma resection), preoperative MRCP assessment of bile duct anomalies is recommended. Second, intraoperatively, the hepatoduodenal ligament should be thoroughly dissected, using “skeletonization” techniques to separate the common bile duct from the portal vein, with dual confirmation of anatomical landmarks prior to division. Furthermore, animal studies indicate that bile duct pressure peaks within 7 days after common bile duct ligation (24), suggesting the need for close monitoring of drainage characteristics and bilirubin levels in the early postoperative period. For established biliary fistulas, the surgical repair combined with drainage approach used in this case aligns with most recommended strategies. However, individualized management should be emphasized, with alternatives such as endoscopic stent placement or percutaneous drainage being viable options (25). Future surgeries should prioritize bile duct protection by incorporating intraoperative cholangiography and cholangioleak monitoring into standard protocols.

Overall, ALPPS implementation in pediatric patients remains challenging due to technical complexity and postoperative complication risks. Nevertheless, with advancing techniques and accumulated experience, ALPPS holds promise as a significant treatment option for pediatric hepatoblastoma, particularly for patients ineligible for liver transplantation or with poor chemotherapy response. Management of biliary fistulas following hepatectomy requires individualized strategies tailored to each patient’s condition, with early diagnosis and intervention being critical for improved outcomes. Future research should further explore preventive strategies for biliary fistulas and more effective minimally invasive treatment approaches to reduce the incidence of postoperative complications.

## Data Availability

The raw data supporting the conclusions of this article will be made available by the authors, without undue reservation.
